# Family caregivers of patients with Head and Neck Cancer seen at an oncologic outpatient service of a Brazilian public university: A qualitative study on reports regarding interpersonal emotional handling

**DOI:** 10.1192/j.eurpsy.2024.661

**Published:** 2024-08-27

**Authors:** S. A. Silva, E. R. Turato, C. S. P. Lima, R. S. E. Sant’Ana, L. S. Valladão, A. C. O. Bispo, C. G. Santos, J. R. P. D. L. Rodrigues, M. R. Suedt

**Affiliations:** ^1^Lab of Clinical-Qualitative Research, University of Campinas, Campinas, Brazil

## Abstract

**Introduction:**

Humanistic studies applied to the health-illness clinic go beyond explaining cause-effect relationships among disease phenomena, treatments, and preventions. Qualitative research aims to understand symbolic relationships built in life experiences among the manifestations and the people. How to act in front of a person whose physical appearance and odour can be unpleasant, such as in the HNC - Head Neck Cancer? Or whose life history may have been marked by deviant behaviours and negligence in self-care?

**Objectives:**

To interpret emotional meanings attributed through open interviews conducted with relatives about the domestic care of patients with HNC under clinical treatment.

**Methods:**

Sample composed of family caregivers of patients with HNC, sent sequentially by colleagues from the clinical service who were informed of the research. The study used the Clinical-Qualitative Method (Turato. Portuguese Psychos. J, 2000 2(1): 93-108). Semi-Directed Interview with Open-ended Questions In-Depth and Field Notes was used for data collection. The employ of the Seven Steps of the Clinical-Qualitative Content Analysis (Faria-Schützer et al. Cien Saude Colet. 2021; 26(1): 265-274) has permitted the understanding of the topics. Sample closed with 12 persons according to the information saturation strategy (Fontanella et al. Cad Saude Publica. 2008; 24(1): 17-27), conducted by the first author, a female psychologist. To interpret the empirical material, we use Medical/Health Psychology, the psychodynamics of relationships of the Balintian framework, disease and illness while modes of un-health, psychic defence mechanisms against anguish. Validation by peers from the Lab of Clinical-Qualitative Research Laboratory, at the State University of Campinas.

**Results:**

For this presentation, we listed three categories from the free-floating re-readings: (1) Certain need to recognize the care provided as a handling strategy with effort, putting in this ‘validation’ their relief regarding natural suffering of the care process; (2) Caregiver’s psychological fantasies of omnipotence in the care process, frequently perceiving the reality a phenomenologically and necessarily distorted by the caregiver. (3) Moments of impotence feeling in front of the finitude reality that it knows will arrive.

**Conclusions:**

The family caregivers can present certain emotional defences, such as subtle magical thinking, in which they distort the reality experienced as a management strategy and validation of their care. They act so to alleviate their psychological and existential suffering. Group meetings with family members to talk openly about the difficulties on the psychological management of patients with HNC, coordinated by a psychotherapist, are effective as a space for creativity in daily management at home and a space for catharsis.

**Disclosure of Interest:**

None Declared

